# Approaches to the Potential Therapy of COVID-19: A General Overview from the Medicinal Chemistry Perspective

**DOI:** 10.3390/molecules27030658

**Published:** 2022-01-20

**Authors:** J. Carlos Menéndez

**Affiliations:** Organic and Medicinal Chemistry Unit, Department of Chemistry in Pharmaceutical Sciences, Faculty of Pharmacy, Universidad Complutense, 28040 Madrid, Spain; josecm@ucm.es

**Keywords:** SARS-CoV-2, S antibodies, serine protease inhibitors, 3CLpro inhibitors, RdRp inhibitors, JAK-STAT inhibitors, corticoids, SphK2 inhibitors, IL6 antibodies

## Abstract

In spite of advances in vaccination, control of the COVID-19 pandemic will require the use of pharmacological treatments against SARS-CoV2. Their development needs to consider the existence of two phases in the disease, namely the viral infection and the inflammatory stages. The main targets for antiviral therapeutic intervention are: (a) viral proteins, including the spike (S) protein characteristic of the viral cover and the viral proteases in charge of processing the polyprotein arising from viral genome translation; (b) host proteins, such as those involved in the processes related to viral entry into the host cell and the release of the viral genome inside the cell, the elongation factor eEF1A and importins. The use of antivirals targeted at host proteins is less developed but it has the potential advantage of not being affected by mutations in the genome of the virus and therefore being active against all its variants. Regarding drugs that address the hyperinflammatory phase of the disease triggered by the so-called cytokine storm, the following strategies are particularly relevant: (a) drugs targeting JAK kinases; (b) sphingosine kinase 2 inhibitors; (c) antibodies against interleukin 6 or its receptor; (d) use of the traditional anti-inflammatory corticosteroids.

## 1. Introduction

Vaccines are the main method for the prevention of viral diseases, and the power of this approach has been recently exemplified by the speed of the development of COVID-19 vaccines [1]. Vaccine availability notwithstanding, it is of the greatest importance to also have access to antiviral pharmacological treatments to cure the disease if it becomes established. Indeed, for some viruses such as HIV, no effective vaccines have been marketed so far, but nevertheless the advances made in anti-HIV drugs have allowed a fatal disease to be turned into a chronic one. Thus, the pharmacological treatment of the COVID-19 disease would be complementary to vaccination, leading to a strategy similar to the one used in the case of influenza. In the specific case of COVID-19, there are several additional reasons that make imperative the development of pharmacological treatments:

1. Currently known vaccines are not 100% effective, nor do they fully prevent the transmission of the disease.
2. Worldwide vaccine distribution faces formidable logistic obstacles that have rendered it rather slow, especially in underdeveloped countries.
3. It is necessary to treat those patients that develop the disease in spite of being vaccinated.
4. A pharmacological treatment would lead to more benign symptoms and therefore the saturation of health systems by COVID-19 patients would be less likely.
5. Pharmacological treatments can be designed to address multiple targets that are highly conserved among variants of the virus, or host-related targets. Thus, it has the potential to enable antiviral activity for all the viral variants.

**Figure 1:**
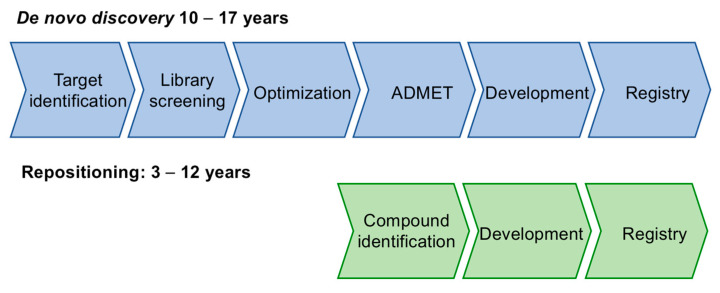
The drug repositioning process.

The traditional de novo drug discovery process is highly complex and requires an average of about 15 years to bring a molecule to the market. Due to the urgency of finding treatments for COVID-19, for the moment, research efforts have been focused on the alternative strategy, known as repositioning. This method involves using a previously known drug for a new indication, and can reduce drastically the time required for the drug discovery and development process (Figure 1).

**Figure 2:**
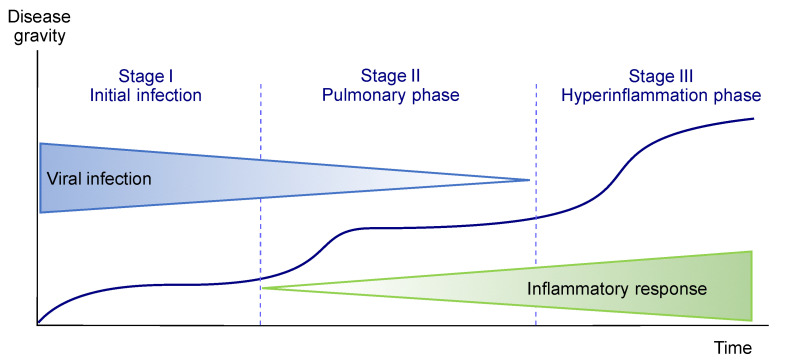
Stages in the evolution of the COVID-19 infection.

The temporal course of the COVID-19 infection has some peculiarities that should be taken into account when choosing a treatment. In the initial stages of the viral infection, symptoms are mild and include fever, myalgia and dry cough. At more advanced stages of the disease, pulmonary symptoms such as dyspnea and hypoxia appear. Although the viral load is diminishing, the condition of some patients worsens because of their own immune response. Thus, life-threatening COVID-19 disease is often associated to the cytokine release syndrome (CRS), i.e., a systemic inflammatory response that occurs when large numbers of white blood cells release inflammatory cytokines (“cytokine storm”), which in turn activate additional white blood cells (Figure 2). As a consequence, COVID-19 therapy should be addressed at the virus itself during early stages of the disease and to the exacerbated inflammatory response at late stages [2].

One final remark regarding COVID-19 is that clinical experience shows it to be a very heterogeneous disease and that only clinical assays involving large numbers of patients give reliable information. Unfortunately, many of the known clinical assays, especially those performed in the early stages of the pandemic, do not comply with this requirement, potentially leading to erroneous conclusions regarding the usefulness of candidate molecules.

Due to the huge importance of the COVID-19 challenge worldwide, a large number of molecules have been assayed in vitro, and over 500 pharmacological treatments have undergone clinical assays [3]. We will discuss the most relevant ones in the following sections, using the molecular mechanism of action as the organizing principle. Criteria for compound inclusion were: (a) small-molecule or biological drugs that have been approved or were considered to be reasonably close to approval at the time of writing (late December 2021); (b) compounds that act by a particularly relevant mechanism that was considered likely to yield approved drugs in the future. These criteria provide a different perspective from previous reviews [4,5,6,7] describing the medicinal chemistry perspective towards the development of pharmacological treatments for COVID-19.

## 2. Structure of the SARS-CoV2 Virus

**Figure 3:**
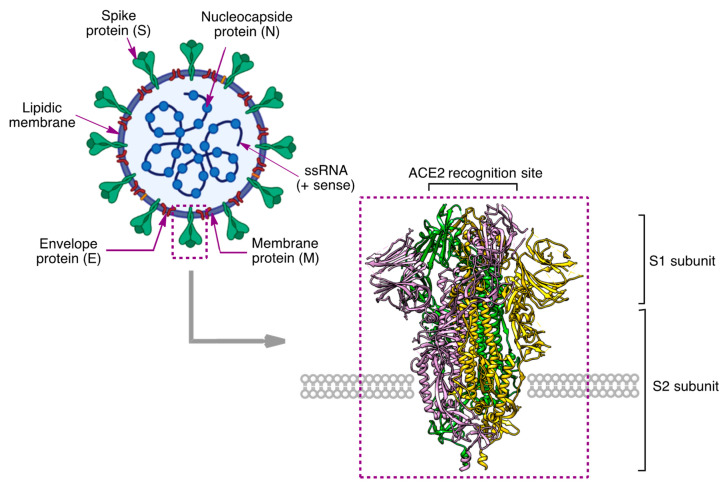
Schematic structure of the SARS-CoV2 virus and its prefusion spike protein (pdb 6VXX).

The SARS-CoV2 (severe acute respiratory syndrome coronavirus 2) virus belongs to the *Coronaviridae* family and is a positive-strand RNA virus. Its genome comprises around 30,000 nucleotides and contains four genes, which codify the surface protein characteristic of coronaviruses (Figure 3) [8,9]:

1. Glycoprotein S, which exists as a homotrimer and forms the characteristic spikes found in the viral surface. Acting as a fusion protein, it allows entry of the virus into the host cell following recognition by its ACE2 membrane protein.
2. Envelope protein (E), which forms the virus cover.
3. Membrane protein (M), which forms the matrix that connects the cover with the inner part of the virus.
4. Nucleocapsid (N) phosphoprotein, which holds the viral genome, a piece of positive-strand RNA.

It also comprises the non-structural open reading frames that codify non-structural proteins such as viral enzymes needed for its intracellular replication.

The viral spike (S) glycoprotein is particularly important because it plays a key role in receptor recognition and cell membrane fusion. It contains 1273 amino acids, which form a signal peptide at the N-terminus (residues 1–13), the S1 subunit (residues 14–685), responsible for receptor binding, and the S2 subunit (residues 686–1273), involved in membrane fusion. Following the outbreak of COVID-19, the in vitro structures of the SARS-CoV-2 S protein in the prefusion state were solved by X-ray crystallography [10] (Figure 3), cryo-electron microscopy [11], and other techniques. Cryo-electron tomography allowed the in situ structure of the postfusion spike at nanometer resolution to be solved [12].

## 3. Antiviral Therapy

### 3.1. The Viral Multiplication Cycle and Associated Drug Targets

**Figure 4:**
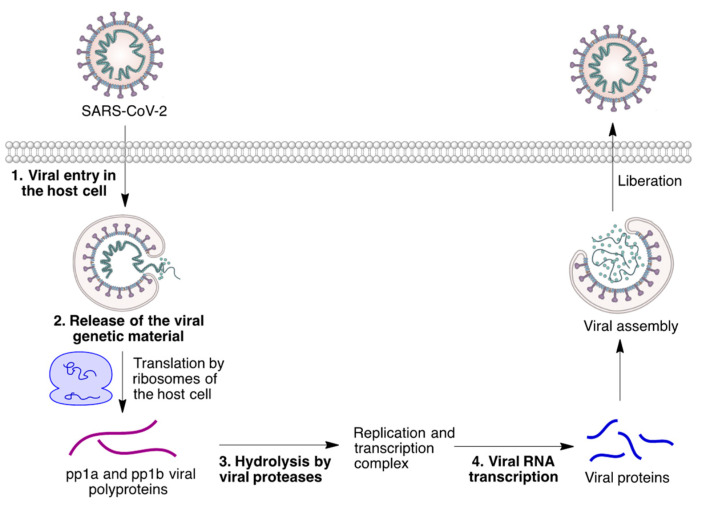
A schematic summary of the cell multiplication cycle of the SARS-CoV2 virus. The main sites for the action of drugs discussed in this review are shown in bold.

The SARS-CoV2 multiplication cycle is summarized in Figure 4, and can be summarized as follows. The proteins involved in these processes are potential targets for drug discovery in this area.

1. The S glycoprotein at the virus cover interacts with angiotensin converting the enzyme (ACE2) at the host cell membrane. This interaction triggers viral entry into the host cell by endocytosis.
2. The endosome that contains the viral particle is fused to a lysosome. The consequent pH reduction promotes the fusion of the viral and endosome membranes, releasing the viral nucleocapsid into the host cell cytoplasm.
3. Proteases from the host cell degrade this capsid, liberating the viral RNA.
4. Because it is a positive-strand RNA, it is able to be directly translated by the host cell ribosomes. This leads to the synthesis of viral polyproteins.
5. These polyproteins are processed by viral proteases, forming the replication and transcription complex.
6. The complementary negative-strand RNA that serves as a template for the replication of the viral genome is next synthesized. Moreover, the replication and transcription complex also gives rise to a positive-strand subgenomic RNA, whose translation furnishes the viral proteins.
7. The viral components are assembled in the endoplasmic reticulum, and a fragment of the ER membrane is used as the viral membrane.
8. The new viral particle travels to the surface of the cell, employing the cell system for vesicle transport.
9. After a final maturation process, with the participation of viral proteases, the infectious viral particle is released by exocytosis and can infect a fresh host cell.

### 3.2. Drugs Acting on the Spike Protein or the Viral Entry Process

At the host cell membrane, infective viral particles bind to the angiotensin converting enzyme (ACE), which acts as its main receptor. Then, a serine protease known as TMPRSS2 (transmembrane serine protease 2) and other host serine proteases hydrolyze the viral protein S at two sites (S1/S2 and S2′), allowing the fusion of both membranes and initiating the viral entry process by endocytosis.

**Figure 5:**
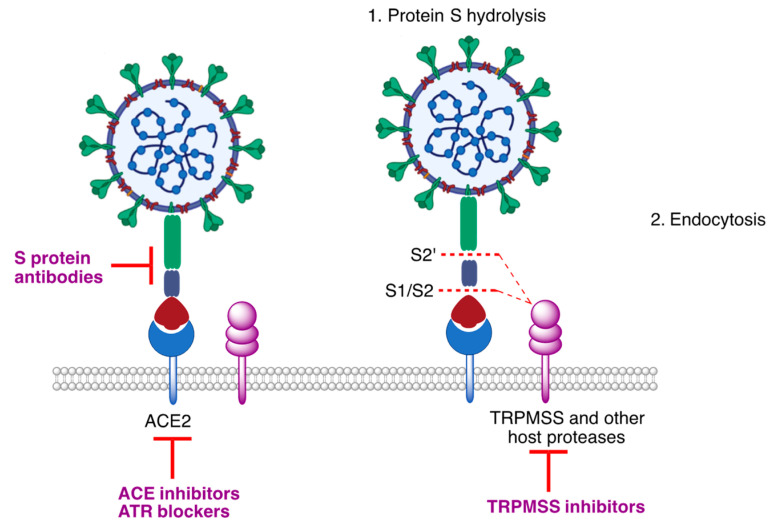
The initial stages of the entry process of SARS-CoV2 virus to the host cell and some points of drug action.

Several of the proteins acting in this process are suitable potential targets for antiviral drugs, including the spike protein characteristic of SARS-CoV-2, the ACE-2 receptor and the TMPRSS2 protease (Figure 5).

#### 3.2.1. Antibodies against the Spike Glycoprotein

The spike glycoprotein is a suitable target for neutralizing antibodies, many of which have been approved in recent months for COVID-19 treatment, reaching an unprecedented pace of approval. These include several molecules from Eli Lilly such as bamlanivimab (LY-CoV555) and etesevimab (LY-CoV016 or JS016); from Regeneron, casirivimab (REGN10933) and imdevimab (REGN10987) (their combination has been marketed as REGEN-COV); from AstraZeneca, cilgavimab (CoV2-2130 or AZD1061) and tixagevimab (CoV2-2196 or AZD8955) (their combination has been marketed as Evusheld); from GSK and Vir Biotechnology, sotrovimab (VIR-7831, Xevudy) [13]; from Celltrion, regdanvimab (CT-P59, Regkirona). Many others are in Phase 3 clinical studies and some of them will undoubtedly be approved soon [13]. The use of antibodies from convalescent patients has also been assayed, especially during the first wave of the disease. Furthermore, the cilgavimab/tixagevimab combination has been recently approved by the FDA for pre-exposure prevention of COVID-19 in certain individuals [14]. These antibodies are targeted at either the receptor-binding domain (RBD) or the N-terminal domain (NTD) of the S-glycoprotein.

The use of these antibodies leads to a somewhat paradoxical situation, since they are primarily indicated at early stages of the disease, when patients do not require hospitalization, but they need to be administered intravenously, which requires hospital settings. Currently, antibody intramuscular or subcutaneous administration is being investigated in order to solve this issue. Another problem hampering the generalized use of antibodies in COVID-19 therapy is their very high cost. Even more problematic is the recent finding that the known antibodies do not act on all variants of the virus. More specifically, the activity of 17 of 19 antibodies tested, including the ones authorized for therapeutic use in patients, were either impaired or completely abolished in the Omicron variant [15].

#### 3.2.2. Inhibitors of the S-glycoprotein—ACE-2 Interaction

Umifenovir (arbidol) is a broad-spectrum antiviral, developed in the Soviet Union in the 1970s and approved in China and Russia against influenza. More recently, its activity against SARS-CoV-2 has been demonstrated. Besides the additional mechanisms associated to its membrane tropism, this compound is able to hamper the protein–protein interaction between the viral S-glycoprotein and the host cell ACE-2, by binding to the recognition domain of the latter [16]. Further details of the use of this compound in COVID-19 are given in Section 3.3.2 below.

#### 3.2.3. ACE Inhibitors and ATR Blockers

**Figure 6:**
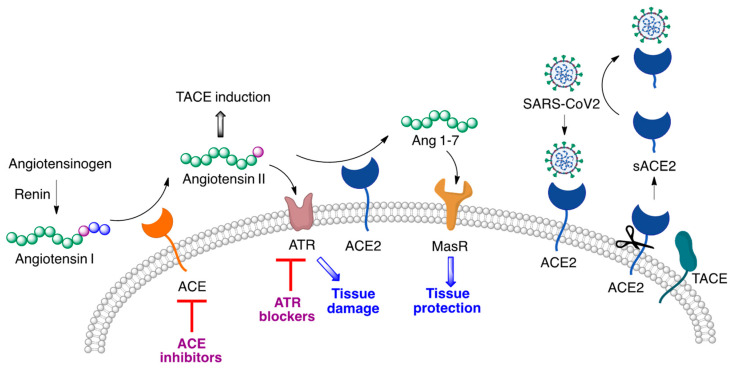
Two pathways explaining the potential beneficial effects of angiotensin-converting enzyme inhibitors and angiotensin receptor blockers in COVID-19 patients.

Due to the fact that it is the cell receptor to which the viral spike protein binds, ACE has been investigated as a potential target for COVID-19 therapy. Inhibitors of this enzyme are well-known antihypertensive agents, and angiotensin receptor antagonists are also widely used for this indication. These antihypertensive drugs have been described to increase the expression of the ACE2 enzyme, the SARS-CoV2 cell entry receptor, and for this reason it was speculated during the early stages of the COVID-19 pandemic that this class of drugs might facilitate infection. On the other hand, it has been argued that this increased ACE2 expression could well be protective and diminish lung injuries [17]. One reason is that the renin–angiotensin system involves, on one hand, the biosynthesis of angiotensin II by ACE-mediated hydrolysis of angiotensin I, and on the other hand its degradation to angiotensin 1–7 by ACE2. The latter peptide has an anti-inflammatory, protective response through the activation of the Mas receptor, which counters the inflammatory response associated with activation of the angiotensin II receptor. Furthermore, angiotensin II induces the TNF-α converting enzyme (TACE), leading to cleavage of ACE2 and generation of soluble ACE2 (sACE2), whose levels are significantly increased in hypertensive patients treated with ACE inhibitors or angiotensin receptor (ATR) blockers, This soluble protein serves as a decoy target for the viral particles, preventing their attachment to the host cells (Figure 6).

**Figure 7:**
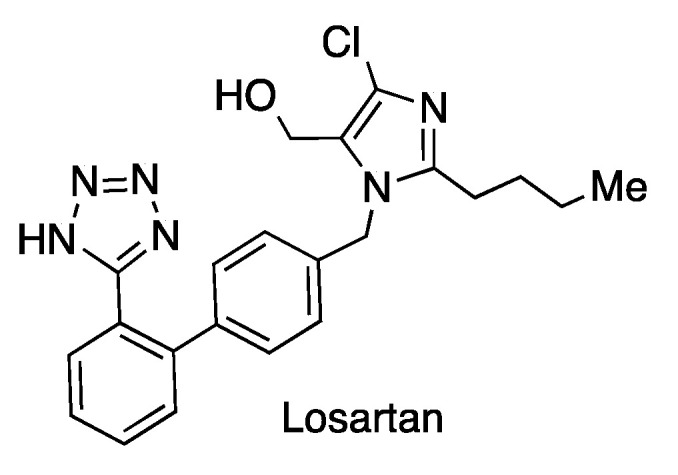
Structure of losartan, an angiotensin receptor blocker.

A number of clinical trials have been initiated with angiotensin receptor antagonists against COVID-19 [18]. The best-studied one is losartan (Figure 7), which unfortunately showed no reduction in the hospitalization time of patients with moderately serious disease [19].

There is another aspect of interest related to the use of ACE inhibitors or ATR blockers in COVID-19 patients. Due to reports showing that inhibitors of the renin–angiotensin system, including both classes of drugs, might increase ACE2 expression, there were initial concerns that they might influence the severity of COVID-19. Several clinical studies have investigated this possibility, concluding that renin–angiotensin system inhibitors can be safely continued in patients hospitalized with COVID-19 [20].

#### 3.2.4. TMPRSS2 Inhibitors

Following binding to host cells via ACE2, virus entry requires that the S protein is cleaved by proteases in the host cell surface, most notably TMPRSS2 and furin; cathepsins are also relevant because of their role in the lysosomal pathway of viral activation.

**Figure 8:**
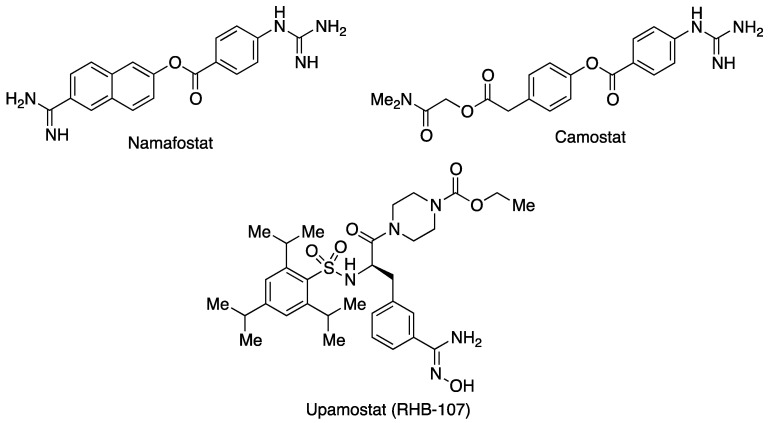
Some inhibitors of the human TMPRSS2 enzyme.

TMPRSS2, a serine protease, is a better potential target for COVID-19 treatment than furin, which has clearer physiological roles [21]. During the early stages of the first wave of the disease, nafamostat, a serine protease inhibitor with activity on TMPRSS2 from Katsura Chemical Co, was explored for the treatment of COVID-19 pneumonia in elderly patients needing oxygen therapy [22]. The structurally related camostat (Figure 8) is another inhibitor of the same class of proteases that has been in clinical use in Japan for many years for the treatment of chronic pancreatitis and reflux esophagitis and manufactured by Ono Pharmaceutical under the trade name Foipan. It has been the subject of several Phase 1 and 2 clinical assays against COVID-19, alone or in combination with other agents, although it did not lead to an effect significantly better than placebo in terms of hospitalization time or disease gravity [23]. Additional clinical studies are ongoing and will shed light on the usefulness of this drug. Upamostat (RHB-107, Mesupron), from Red Hill BioPharma, is another inhibitor of serine proteases that displays oral activity and is under clinical study for the treatment of COVID-19 [24].

### 3.3. Drugs That Prevent the Viral Genome Release

**Figure 9:**
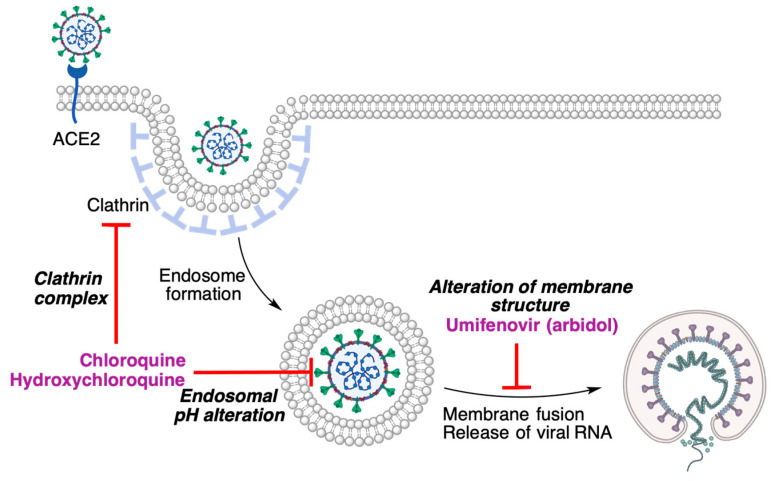
The viral genome release process and some of its inhibitors.

Following recognition by the ACE2 receptor, a clathrin-mediated endocytosis process allows the virus to enter the host cell [25]. Once inside the endosome, pH lowering due to fusion with cell lysosomes promotes the fusion of the viral and endosomal membranes, liberating the viral nucleocapsid inside the cell. This process may be altered by drugs that interact with clathrin, alter the endosomal pH or interfere with membrane fusion (Figure 9).

#### 3.3.1. Chloroquine and Hydroxychloroquine

**Figure 10:**
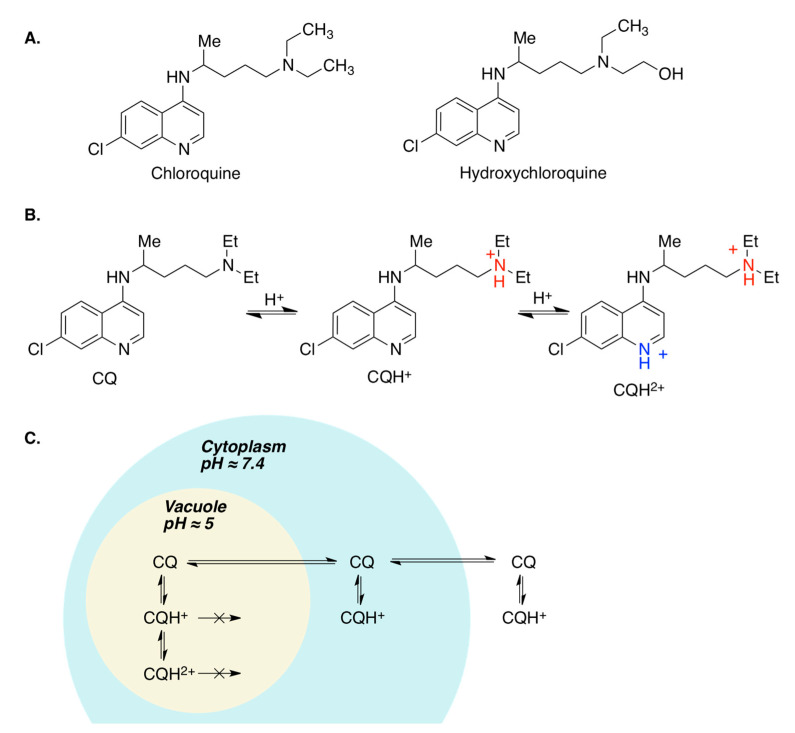
(**A**) Structures of chloroquine and hydroxychloroquine. (**B**) Protonation equilibria in the chloroquine molecule. (**C**) Retention of chloroquine in acidic vesicles.

The antimalarial drug chloroquine (CQ) and its metabolite hydroxychloroquine (HCQ) have shown in vitro activity against several viruses, including SARS-CoV2 [26]. Although additional mechanisms are involved, including clathrin interaction, their antiviral activity seems to be mainly due to their ability to alter endosomal pH [27]. These compounds have two basic centers, namely the heterocyclic nitrogen belonging to the 4-aminoquinoline moiety, and the tertiary amine at the end of the side chain. As a consequence, at acidic pH values they can generate a monoprotonated species (CQH^+^) and even small amounts of a diprotonated one (CQH^2+^), which, due to their low lipophilicity, become trapped in the acidic organelles, raising vesicle pH and hampering the membrane fusion process (Figure 10). Moreover, both drugs have immunomodulatory effects, and indeed HCQ is used to treat certain autoimmune diseases such as rheumatoid arthritis and systemic lupus erythematosus [28]. This property of CQ and HCQ renders them potentially useful for suppressing the immune system response characteristic of the severe forms of COVID19. Both chloroquine and its hydroxy derivative were broadly used in the clinic in many countries during the first wave of COVID-19. However, subsequent clinical studies have not shown evidence of clinical benefit and safety in their use for treating the SARS-CoV2 infection [29,30,31].

#### 3.3.2. Umifenovir (Arbidol)

**Figure 11:**
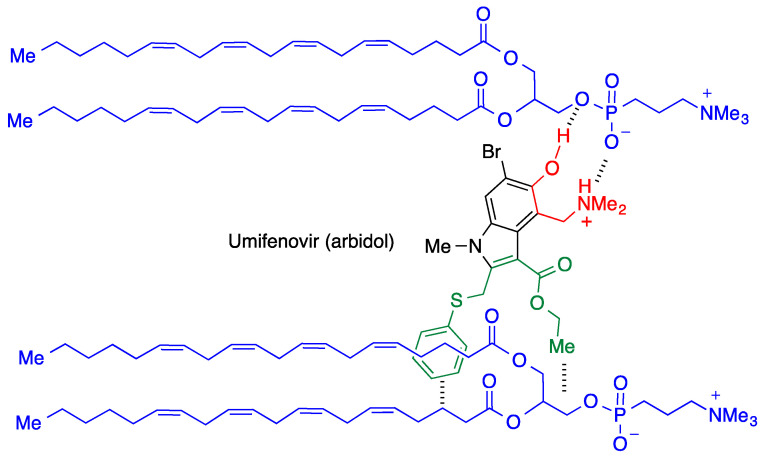
Interaction of umifenovir with cell membrane phospholipids.

This drug, described above as an inhibitor of the S1—ACE-2 interaction, is a broad-spectrum antiviral with a high affinity for the host cell membranes. This is due to its ability to interact by ionic and hydrogen bonding interactions of its C4 and C5 substituents with the polar head of phospholipids, while lipophilic parts of the molecule establish hydrophobic interactions with the phospholipid apolar chains (Figure 11). The membrane stabilization thus achieved hampers the membrane fusion process required to release the viral genome [32].

Umifenovir has been clinically assayed in COVID-19 patients, and a recent meta-analysis has revealed that it improves the response to the lopinavir/ritonavir combination, although when used on its own, it is not statistically different from the absence of treatment [33].

### 3.4. Inhibitors of the Viral Protease

Once the viral genetic material has been released and translated by the ribosomes of the host cell, the resulting polyprotein needs to be hydrolyzed to yield the viral structural and functional proteins, including the RNA-dependent RNA polymerase (RdRp). This hydrolytic step is catalyzed by two viral proteases, namely the 3C-like protease (3CLpro) and the papain-like protease (PLpro).

**Figure 12:**
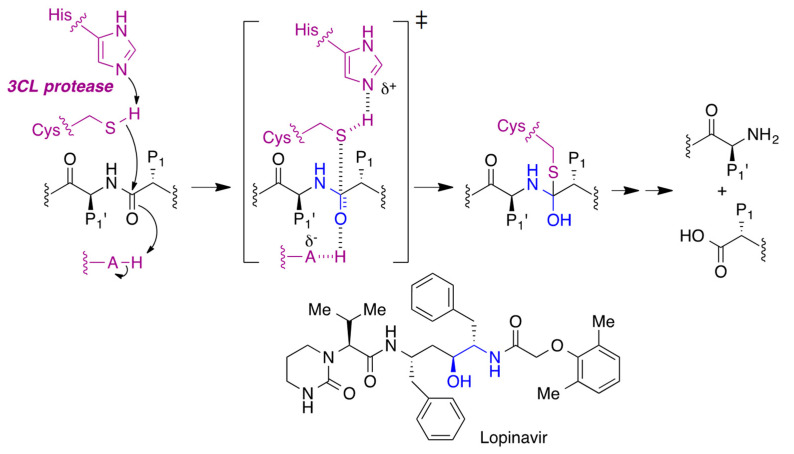
Inhibition of the viral 3CL protease by lopinavir.

Lopinavir, initially developed as an anti-HIV drug, inhibits the 3C-like protease [34]. This enzyme belongs to the cysteine protease family, and its inhibition takes place thanks to the analogy of the drug with the transition state of the hydrolysis reaction (Figure 12).

**Figure 13:**
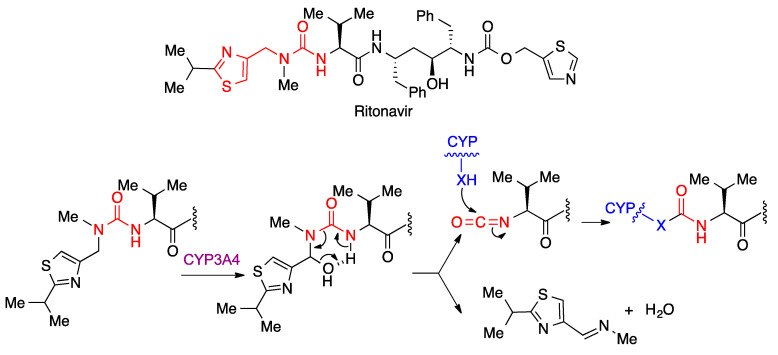
Ritonavir as a pharmacokinetic enhancer of lopinavir.

Due to the poor metabolic stability of lopinavir, which is a substrate of the CYP3A4 cytochrome, it is generally associated to ritonavir, which is a suicide cytochrome inhibitor that acts by metabolic generation of an isocyanate intermediate that then carbamoylates a nucleophilic residue at the cytochrome (Figure 13).

The lopinavir-ritonavir combination (Kaletra or Aluvia) was widely employed during the first wave of the COVID-19 pandemic, but subsequent clinical trials have shown that its addition to the standard care provides no benefit to patients [35].

**Figure 14:**
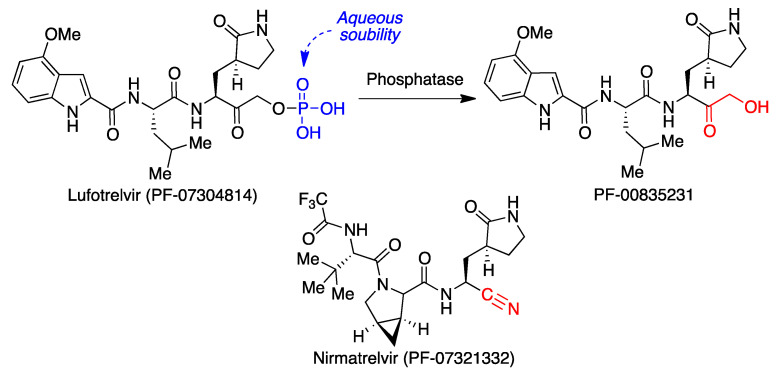
Structures of lufotrelvir, PF-00835231 and nirmatrelvir.

Lufotrelvir (PF-07304814), from Pfizer, is a water-soluble phosphate prodrug suitable for intravenous use whose active form is the 3CLpro inhibitor PF-00835231 (Figure 14). This compound was initially developed during the 2002–2003 SARS-CoV outbreak and is a broad-spectrum inhibitor of coronavirus proteases. It was approved by the FDA for emergency use against COVID-19, but it was soon superseded by its orally active analogue nirmatrelvir (PF-07321332), also from Pfizer, which, in combination with ritonavir, was granted emergency use by the FDA in December 2021 for COVID-19 treatment under the trade name Paxlovid. The nirmatrelvir-ritonavir association seems highly promising, since it has shown an efficacy of about 88% against hospitalization or death in adult outpatients after its administration within 5 days of the onset of COVID-19 symptoms. Due to the presence of the cytochrome inhibitor ritonavir, potentially serious interactions with other drugs may take place and need to be considered.

**Figure 15:**
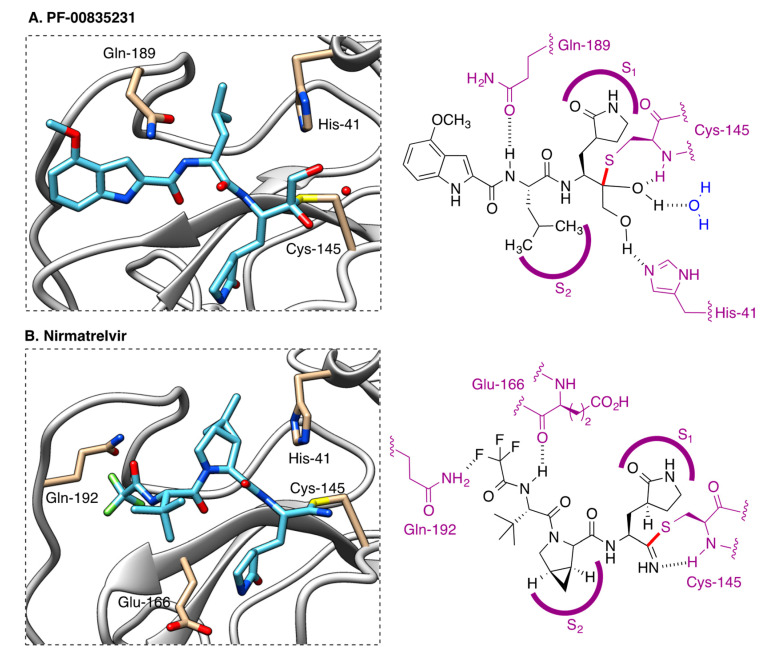
Interaction of the SARS-CoV2 3CL protease with: (**A**) PF-00835231 (pdb 6XHM). (**B**) Nirmatrelvir (pdb 7VH8).

Both PF-00835231 and nirmatrelvir have been shown to interact covalently, although reversibly, with the key Cys-145 residue of the viral protease. In the case of PF-00835231 [36], the Cys mercapto group forms a covalent bond with the ketone group of the terminal α-hydroxyketone moiety in the drug to yield a hydrogen bond-stabilized hemithioacetal, together with additional hydrogen bonds with a number of other key active site amino acids (Figure 15A). Nirmatrelvir [37] occupies the active site in a similar way, with Cys-145 binding covalently to its nitrile group via a Pinner-like reaction and several hydrogen bonds reinforcing the interaction (Figure 15B).

### 3.5. Antivirals That Prevent Viral Genome Replication

#### 3.5.1. General Aspects

**Figure 16:**
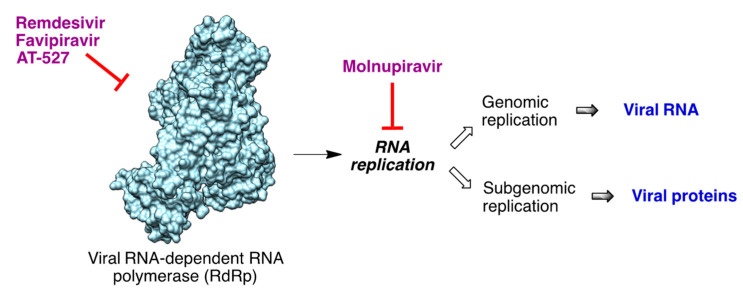
Processes involved in SARS-CoV2 RNA replication. The structure of the RdRp enzyme was generated from pdb 6M71.

One of the proteins arising from the translation of the viral RNA liberated in the host cell cytoplasm is the RNA-dependent RNA polymerase (RdRp). This enzyme carries out the synthesis of several RNAs, including the viral genomic RNA and several subgenomic RNAs, which are later translated into the viral proteins (Figure 16). Therefore, RdRp is an important potential target in the discovery of drugs against COVID-19 [38,39].

**Figure 17:**
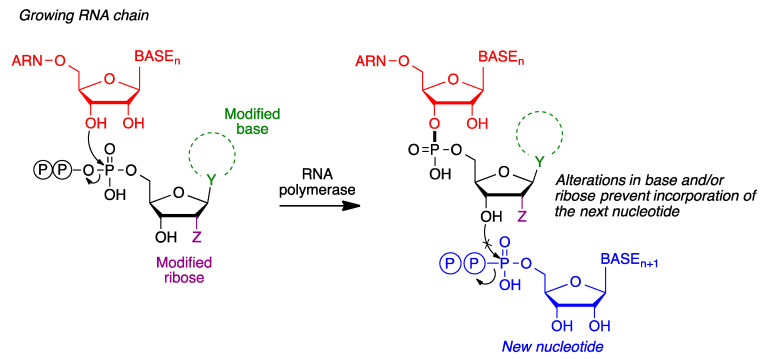
General mechanism of action of nucleoside-type RNA polymerase inhibitors.

RNA polymerase inhibition is a frequent strategy for antiviral drug discovery. Inhibitors of this enzyme are normally modified nucleosides, with structural changes in the nucleobase and/or ribose moiety. Following triphosphorylation by kinases, these compounds can be recognized by RNA polymerase and act as its substrates, thereby becoming incorporated into a growing RNA chain. However, the structural modifications prevent the incorporation of an additional nucleotide and therefore the growth of the RNA chain is interrupted (Figure 17).

#### 3.5.2. Remdesivir

**Figure 18:**
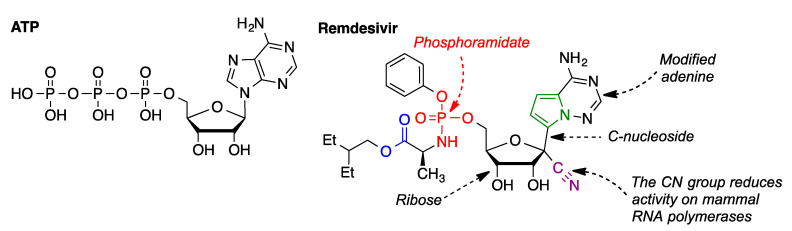
Compared structures of ATP and remdesivir.

Remdesivir (GS-5734, Veklury), from Gilead Sciences, was the first anti-COVID-19 drug that received FDA approval. This compound, after phosphorylation, can be regarded as an analogue of ATP, one of the substrates of the RNA-dependent RNA polymerase. Remdesivir has some structural peculiarities that are underscored in Figure 18. Its adenine fragment is modified, and it is joined to ribose by a C–C bond, rendering the compound a C-nucleoside. The presence of a cyano group at the anomeric carbon is unusual, and it leads to a lower activity on mammal RNA polymerases. Remdesivir is a prodrug, since its phosphorous atom is part of a phosphoramidate function, which needs to be hydrolyzed to the corresponding phosphate.

**Figure 19:**
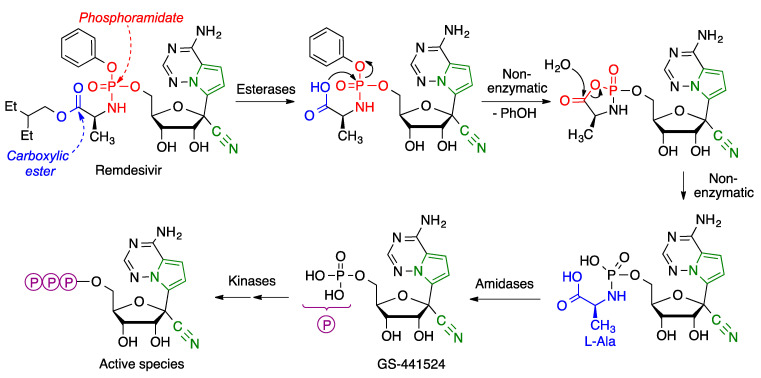
Bioactivation of remdesivir.

Although nucleoside derivatives generally act in di- or triphosphate form, these species are not employed as such because of their high polarity, which hampers their absorption across cell membranes. Phosphoramidates and related prodrugs, developed by McGuigan, are generally described as ProTide (PROdrug + nucleoTIDE), and have found widespread application in recent years [40]. The ionizable groups of these compounds are blocked by lipophilic moieties, allowing a good membrane permeability. The prodrug is activated by the series of reactions summarized in Figure 19. The first step is the hydrolysis of the ester group to the corresponding carboxylic acid. This intermediate, owing to the proximity of the phosphoramidate group, undergoes a non-enzymatic cyclization step, resulting in a mixed anhydride, whose high reactivity prompts its fast hydrolysis to a phosphoramide. This compound is then hydrolyzed by an amidase, yielding the nucleotide GS-441524 as a monophosphate. One advantage of this type of prodrugs over nucleosides is the avoidance of an initial nucleoside phosphorylation, which is often slow. Two final kinase-catalyzed phosphorylation steps furnish the triphosphate required for recognition by the RNA polymerase and stalling of RNA synthesis [41,42].

Due to its mechanism of action, remdesivir is a broad-spectrum agent against monocatenary RNA viral infections. It was initially proposed for the treatment of hepatitis C, and in 2015, it was proved active in *Rhesus* monkeys infected with the Ebola virus. These findings led to its administration to human patients during the Ebola outbreaks that took place in several regions of Africa. However, a 2019, clinical study showed that although the compound had a good safety profile, its activity in Ebola-infected humans was poor. Due to its mechanism of action, it was also proposed as a potential treatment for COVID-19, and after several clinical studies it was the first drug to be approved for this indication (EMA, June 2020; FDA, October 2020, for emergency use). A recent meta-analysis has shown that treatment with remdesivir probably results in a moderate increase in the number of patients recovering from the disease, as well as a moderate decrease in serious adverse events and may also lead to a large reduction in recovery time [43].

#### 3.5.3. Bemnifosbuvir

**Figure 20:**
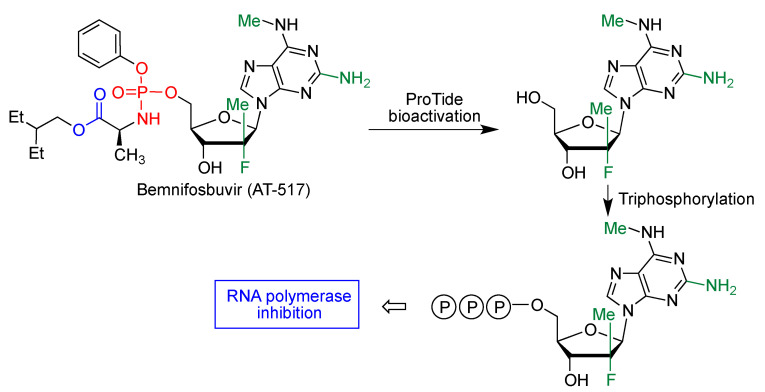
Bioactivation and mechanism of action of bemnifosbuvir.

Another ProTide prodrug, originally designed for the treatment of hepatitis C, is the orally bioavailable bemnifosbuvir (AT-527), developed by Atea Pharmaceuticals and later sold to Roche outside of the United States under the research code RO7496998. Its bioactivation by the above-discussed mechanism yields an adenosine fluoro analogue (Figure 20). Following its triphosphorylation, this compound inhibits SARS-CoV-2 ARN polymerase [44].

Bemnifosbuvir showed good results in early clinical trials but in later stages some inconsistency was observed in the results. For this reason, the planned Phase 3 trials are being redesigned [45].

#### 3.5.4. Favipiravir

**Figure 21:**
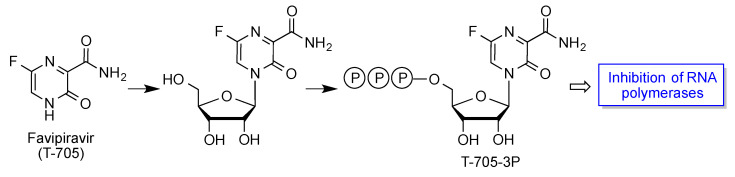
Bioactivation and mechanism of action of favipiravir.

Favipiravir (T-705, Avigan, Avifavir) is another broad-spectrum inhibitor of viral RNA polymerases, which was developed by Toyama Chemical and is commercialized in Japan for the treatment of influenza, having also been assayed against Ebola. It is an analogue of pyrimidine RNA nucleobases and needs to be activated by transformation into its nucleoside triphosphate (Figure 21) [46]. Favipiravir was approved for COVID-19 treatment in Japan, Russia, India and other countries, under emergency provision. A recent systematic review and meta-analysis of clinical trials showed a significant clinical improvement in the favipiravir group vs. the control group during seven days after hospitalization [47].

#### 3.5.5. Other RNA Polymerase Inhibitors

**Figure 22:**
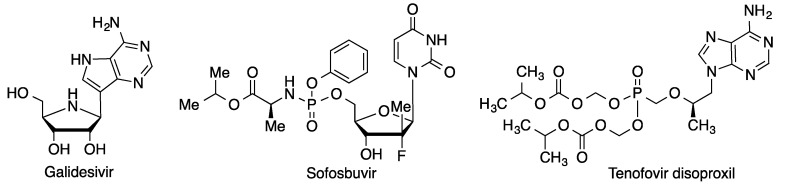
Structures of additional RNA polymerase inhibitors assayed against COVID-19.

Some additional RNA polymerase inhibitors that have been clinically assayed against COVID-19 include galidesivir, from BioCryst, sofosbuvir (Sovaldi) and tenofovir disoproxil (Viread^®^), both from Gilead Sciences and assayed for COVID-19 in combination with other antivirals (Figure 22).

#### 3.5.6. Molnupiravir

**Figure 23:**
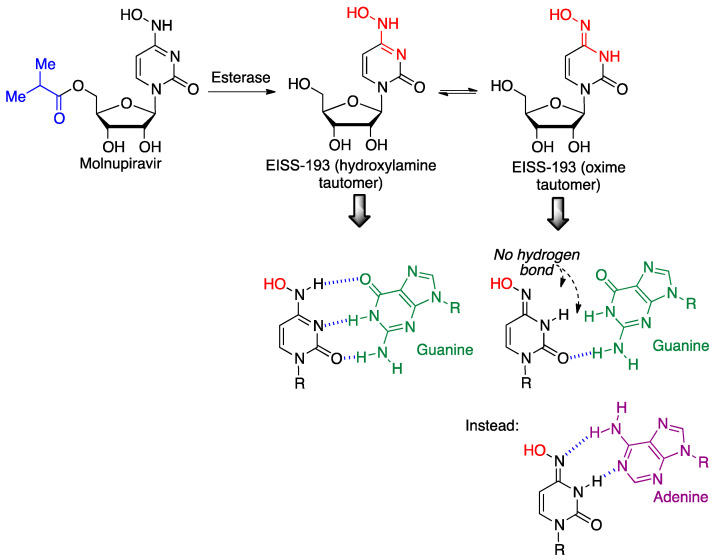
Bioactivation and mechanism of action of molnupiravir.

Molnupiravir (EIDD-2801, Molulife, Molena) is an orally active prodrug of N-hydroxycytidine (EISS-193) developed by Merck, designed to avoid the first-pass effect observed in the active species. N-hydroxycytidine, after its triphosphorylation, can be incorporated into viral RNA chains. The compound can exist in two tautomeric forms, where the hydroxylamine form maintains the usual affinity for guanine, whereas the oxime tautomer loses two of its hydrogen bonds with guanine and tends to bind to adenine instead (Figure 23). This mutation originates errors during the viral RNA replication, a mechanism described as “lethal mutagenesis” [48].

In December 2021, molnupiravir received emergency use authorization from the FDA for the oral treatment of mild-to-moderate coronavirus disease in adults with positive results of direct SARS-CoV-2 viral testing [49], although the full clinical trial results suggest that it is less effective than originally thought in view of the preliminary data. Molnupiravir may cause fetal harm and is therefore not recommended for use during pregnancy. Furthermore, due to its mutation-based mechanism of action, concerns have been expressed over the possibility that its widespread usage may lead to the appearance of new variants of the virus [50].

### 3.6. Antivirals Acting on Host Targets

A therapeutic strategy based on blocking certain host proteins that are essential for viral multiplication would not be susceptible to viral mutations. Furthermore, such treatments would in principle be useful against all variants of the virus. Together with the above-discussed host serine proteases, targeting the proteins mentioned in this Section has these potential advantages. It is interesting to note that the main hit compounds identified in this area are natural products.

#### 3.6.1. Plitidepsin

**Figure 24:**
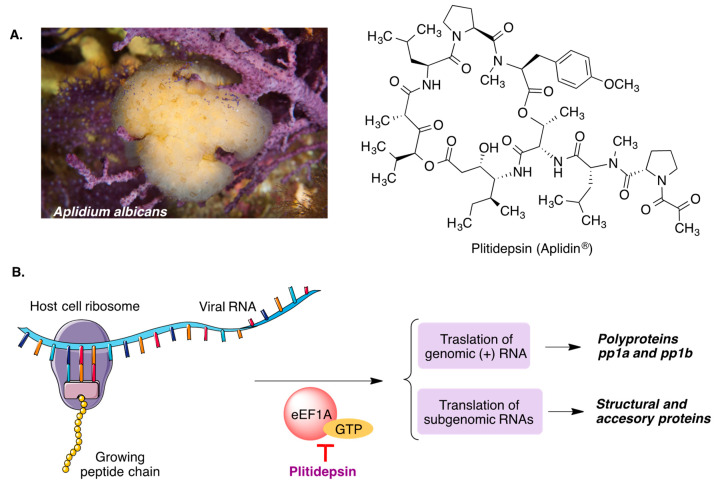
(**A**) Natural source and structure of plitidepsin. (**B**) Antiviral mechanism of action of plitidepsin.

Plitidepsin (Aplidin) is a natural cyclodepsipeptide, first isolated from the marine tunicate *Aplidium albicans* and now obtained by total synthesis. It is being developed by PharmaMar as a treatment for multiple myeloma, and its repurposing for the treatment of COVID-19 has given some promising results. It has been shown that its target is the human elongation factor eEF1A [51], which the SARS-CoV2 virus uses to facilitate the uptake of aminoacyl-tRNAs by the ribosome and is therefore essential for the translation of viral RNA and the subgenomic mRNAs that codify the viral proteins (Figure 24) [52]. Plitidepsin has shown very potent antiviral activity in vitro and is currently under Phase 3 clinical assays. Preliminary results have shown that it is safe and positively influences the outcome of patients hospitalized with COVID-19 [53].

#### 3.6.2. Ivermectin

**Figure 25:**
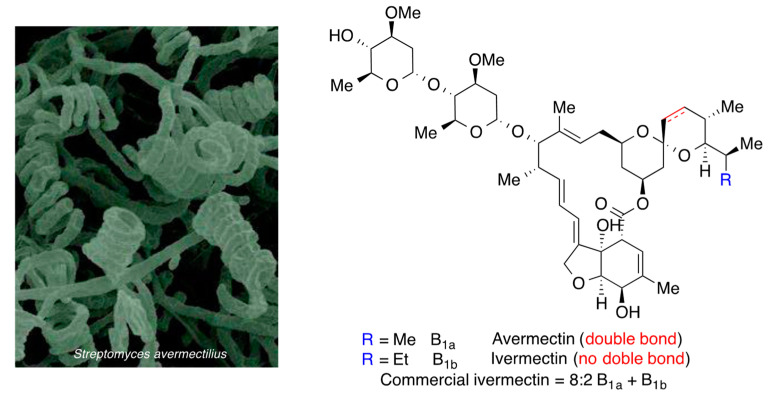
Structure of ivermectin.

Another natural product that has shown antiviral activity related to the blockade of a host cell target is ivermectin, which, together with the closely related avermectins, has an interesting history. The avermectins were discovered by Ōmura in *Streptomyces avermitilis* (currently *avermectinius*) isolated from soil samples and were studied as antiparasitic agents by Campbell in Merck. It was soon found that compounds lacking the double bond at the spirocyclic fragment of the molecule (ivermectins) were less toxic and had a broader antiparasitic spectrum. Commercially available ivermectin is an 8:2 mixture of ivermectins B_1a_ and B_1b_ (Figure 25). It has low toxicity and is widely employed as an antiparasitic medication for veterinary use; it has also been approved for a number of human parasitic diseases, most notably onchocerciasis (river blindness), and also filariasis, strongyloidiasis and pediculosis. Ōmura and Campbell shared half the 2015 Nobel Prize in Physiology/Medicine for their discovery.

Regarding its use against COVID-19, in 2020, ivermectin was discovered to be antiviral in vitro. Its potency was moderate, with an IC_50_ = 2 µM, which is 15–30 times higher than the concentration that can be reached with a dose of 200 µg/kg. Nevertheless, it seemed promising in early clinical testing [54] and it was later found to act on multiple targets, including the viral S protein and the host importins, which act as transporters of several viral proteins (ORF-3a, ORF-6, NSP-1) to the nucleus, where they block the production of the natural antiviral interferon. Moreover, ivermectin was found to act by other mechanisms not based on its viral action, since it is anti-inflammatory and antithrombotic. The clinical efficacy of ivermectin has become highly controversial; one meta-study showed a reduction of the risk of death in COVID-19 patients, especially for avoiding the progression of the disease towards the more severe stages [55] but several problems with the data and the methodology have been pointed out by other authors [56] and another meta-study concluded that the evidence available that can be considered reliable does not support the use of ivermectin for the treatment or prevention of COVID-19 outside of randomized trials [57]. This is also the recommendation of the World Health Organization and of regulatory agencies such as FDA and EMA.

## 4. Drugs That Regulate the Host Immune Response

### 4.1. Inhibitors of the JAK-STAT Signaling Pathway

**Figure 26:**
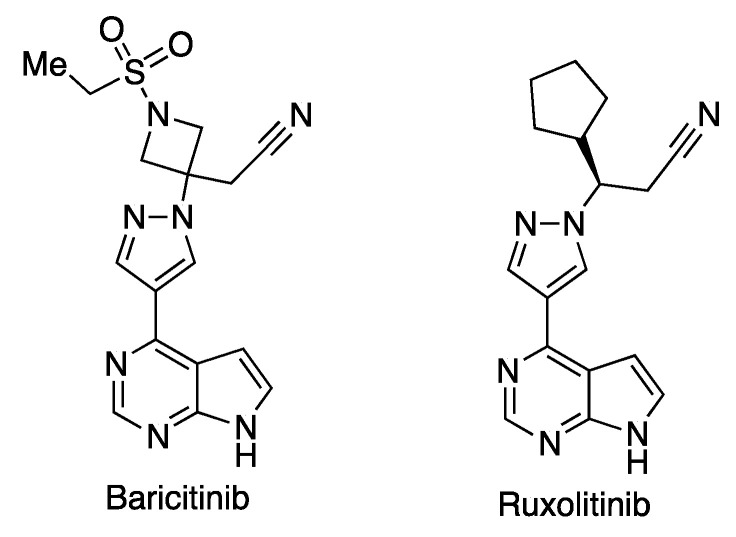
Structures of JAK1/JAK2 kinase inhibitors used against COVID-19.

Baricitinib (Olumiant), from Eli Lilly, is an orally active selective inhibitor of JAK1/JAK2 kinases, approved for the treatment of rheumatoid arthritis due to its ability to regulate the inflammatory immune response [58]. In combination with remdesivir, it has been approved for emergency use in advanced stages of COVID-19. Clinical assays showed that, although treatment with baricitinib did not affect the course of the disease, its addition to the standard treatment led to a reduced mortality in hospitalized adult patients [59]. Moreover, a combination of baricitinib and remdesivir proved to be better than remdesivir alone in reducing recovery time and accelerating improvement in clinical status of patients of advanced COVID-19 [60]. A related molecule, ruxolitinib (Jakafi^®^) (Figure 26), from Incyte Corp in collaboration with Novartis, whose approved use is the treatment of high-risk myelofibrosis, a myeloproliferative disease affecting bone marrow, has been clinically assayed in Phase 3 for COVID-19 patients, but it showed no improvement in terms of the number of patients that evolve to the more advanced stages of the disease.

**Figure 27:**
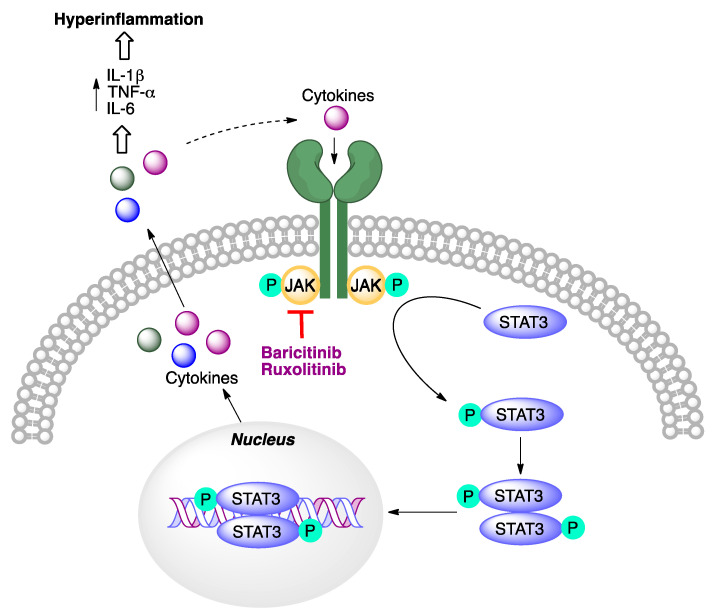
The role of the JAK-STAT pathway in COVID-related hyperinflammation.

The rationale for the use of these molecules for the treatment of COVID-19 is connected to the role of the JAK-STAT signaling pathway in the biosynthesis of cytokines (Figure 27). For this reason, it was hypothesized that its inhibitors would be useful to modulate the immune response of patients and avoid the cytokine storm characteristic of the more severe stages of COVID-19.

### 4.2. Inhibitors of Sphingosine Kinase

**Figure 28:**
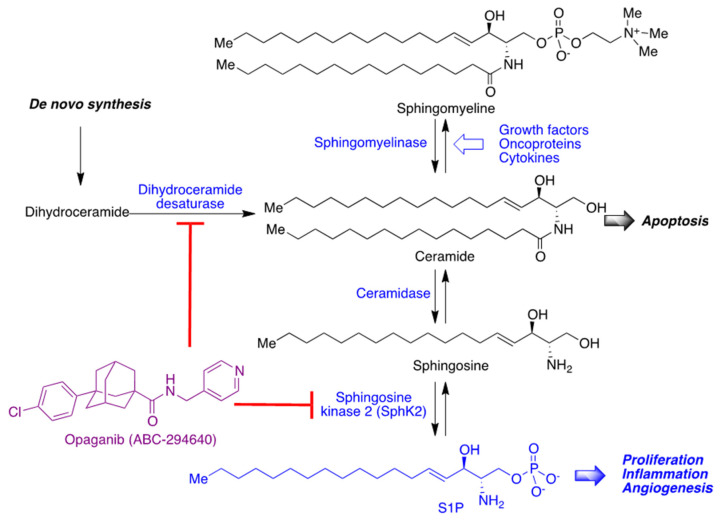
Mechanism of action of opaganib.

Sphingosine-1-phosphate (S1P) activates several signaling pathways and promotes the synthesis of the proinflammatory cytokines TNF-α and IL-6. It also has the capacity to promote the replication of the SARS-CoV-2 virus in vitro. Opaganib (ABC294640, Yeliva), from Red Hill BioPharma, inhibits sphingosine kinase 2 (SphK2), the enzyme responsible for the synthesis of S1P. It also inhibits the previous step in the biosynthetic route, i.e., the transformation of dihydroceramide into ceramide by dihydroceramide desaturase (Figure 28) [61]. Opaganib has been clinically studied in Phase 2/3, showing a good tolerability and activity and a 62% statistically significant reduction in mortality versus the placebo in moderately severe COVID-19 patients [62].

### 4.3. Corticoids

**Figure 29:**
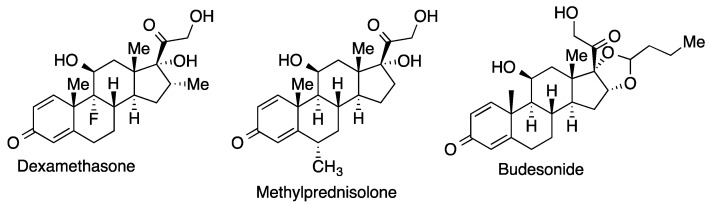
Some corticoids employed in COVID-19 patients.

Dexamethasone, a synthetic corticoid with anti-inflammatory activity, was the first drug proved to improve the survival rate of hospitalized COVID-19 patients under oxygen therapy or forced ventilation. Other corticoids employed for the same indication are methylprednisolone and budesonide (Figure 29). Corticoid treatment is not recommended for patients in less severe stages of the disease, whose mortality rate they seem to increase.

### 4.4. Aviptadil

**Figure 30:**
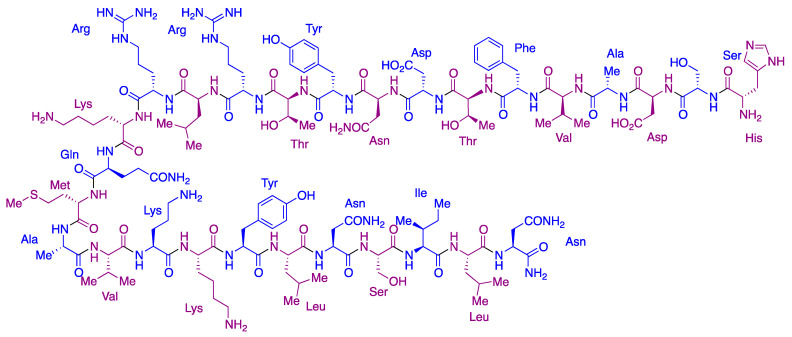
Structure of aviptadil.

Aviptadil (RLF-100, Zyesami, Figure 30) is the vasoactive intestinal peptide (VIP), which binds specifically to the lung endothelium and reduces pulmonary inflammation by reducing the production of proinflammatory cytokines. NRx Pharmaceuticals has performed its clinical study in critical COVID-19 patients [63], finding good safety data, significative improvements in the levels of oxygenation and pulmonary function since the first days of treatment and a highly significant four-fold increased probability of survival. Based on these results, in late December 2021, the company filed a breakthrough therapy designation request for aviptadil in patients at immediate risk of death from COVID-19 despite treatment with other approved therapies.

### 4.5. Antibodies against Interleukin 6 or Its Receptors

**Figure 31:**
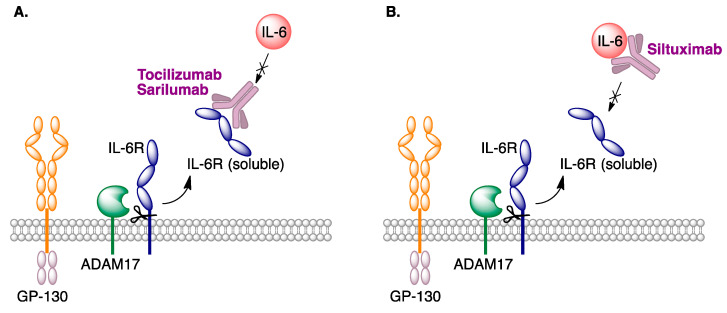
Antibodies targeting: (**A**) Interleukin 6 receptor. (**B**) Interleukin 6.

Interleukin 6 (IL-6) is a proinflammatory cytokine produced by several types of cells, including those of the bronchial epithelium [64]. In late-stage COVID-19 patients, an increase in the levels of this protein is associated to a hyperinflammatory response, and hence the modulation of IL-6 activity is likely to provide a therapeutic option for these patients. The FDA has approved two types of antibodies showing these effects, addressed either at the IL-6 receptor, such as tocilizumab (Actemra), from Genentech, and sarilumab (Kevzara), from Sanofi, or at IL-6 itself, such as siltuximab (Sylvant^®^), from EUSA Pharma (Figure 31). They have been evaluated in patients at the inflammatory phase of the disease, and the following recommendations [65] have been given regarding their use:

1. Tocilizumab, associated to dexamethasone, is recommended for hospitalized patients that show rapid respiratory distress as a consequence of the disease.
2. There is not enough evidence to recommend the use of sarilumab.
3. Siltuximab is not recommended, except in the context of clinical assays.

### 4.6. Other Immunomodulators

Polyphenols are naturally occurring compounds with immunomodulatory activity, and have a potential role in reducing inflammation and preventing the onset of the more serious symptoms of certain chronic diseases. Some clinical trials with polyphenols, most notably quercetin, for the treatment of COVID-19 have been approved, with some interesting results [66].

## 5. Conclusions

In spite of its great importance in the control of the pandemic, the pharmacological therapy of COVID-19 is still in its infancy. One of the main hurdles to overcome is the need to develop treatments in a very short time, which has led to a preference for the drug repurposing approach. Moreover, COVID-19 therapy is conditioned by the existence of two distinct phases in the disease, namely the viral infection and the inflammatory stages. The main viral targets for potential drugs are the viral S protein, host proteins involved in the processes related to viral entry into the host cell and release of the viral genome inside the cell and the viral proteases in charge of processing the polyprotein arising from its translation. The use of antivirals targeted at host proteins, such as serine proteases, the elongation factor eEF1A or importins, has the advantage of not being affected by viral mutations. Regarding drugs that address the hyperinflammatory phase of the disease (cytokine storm), besides the traditional anti-inflammatory corticosteroids, drugs targeting JAK kinases, sphingosine kinase 2 and interleukin 6 seem particularly promising. Regarding future directions for drug discovery in this area, achieving oral therapy is of the utmost importance to relieve pressure on primary medical assistance and hospitals, and it is much more likely to be achieved by small-molecule therapies. Once this goal has been reached through the drug repurposing approach, additional refinements will probably be associated with the use of target-based medicinal chemistry methods. It is to be hoped that research in this area will soon yield safe and efficient anti-COVID-19 drugs that contribute to bring human society back to normality.
